# Atlas of Human Anatomy

**Published:** 1880-12

**Authors:** 


*Atlas of Human Anatomy.*—Published by A. E. Wilde & Co. 51 West Fifth St. Cincinnati, Ohio.

The parts 5 to 14 of this valuable publication are equal to the earlier ones for accuracy of detail and elegance of preparation. It is proposed to complete the work in forty-five parts, each to contain four large plates, mostly life-size. The text will be complete in ten parts of about forty pages, each at the price of fifty cents each part, and will be issued at intervals as the Atlas is published. Four parts of the Atlas will be issued every month, and it is expected that the whole work will be completed about January, 1881.

